# Identifying genetic variants for amyloid β in subcortical vascular cognitive impairment

**DOI:** 10.3389/fnagi.2023.1160536

**Published:** 2023-04-18

**Authors:** Hang-Rai Kim, Sang-Hyuk Jung, Beomsu Kim, Jaeho Kim, Hyemin Jang, Jun Pyo Kim, So Yeon Kim, Duk L. Na, Hee Jin Kim, Kwangsik Nho, Hong-Hee Won, Sang Won Seo

**Affiliations:** ^1^Department of Neurology, Dongguk University Ilsan Hospital, Dongguk University College of Medicine, Goyang, Republic of Korea; ^2^Department of Neurology, Samsung Medical Center, Sungkyunkwan University School of Medicine, Seoul, Republic of Korea; ^3^Alzheimer’s Disease Convergence Research Center, Samsung Medical Center, Seoul, Republic of Korea; ^4^Department of Biostatistics, Epidemiology and Informatics, Perelman School of Medicine, University of Pennsylvania, Philadelphia, PA, United States; ^5^Department of Digital Health, Samsung Advanced Institute for Health Sciences & Technology, Samsung Medical Center, Sungkyunkwan University, Seoul, Republic of Korea; ^6^Department of Neurology, Dongtan Sacred Heart Hospital, Hallym University College of Medicine, Hwaseong, Republic of Korea; ^7^Department of Radiology and Imaging Sciences, Center for Neuroimaging, Indiana University School of Medicine, Indianapolis, IN, United States; ^8^Samsung Genome Institute, Samsung Medical Center, Seoul, Republic of Korea; ^9^Department of Artificial Intelligence, Ajou University, Suwon, Republic of Korea; ^10^Department of Software and Computer Engineering, Ajou University, Suwon, Republic of Korea; ^11^Cell and Gene Therapy Institute, Research Institute for Future Medicine, Samsung Medical Center, Seoul, Republic of Korea; ^12^Department of Health Sciences and Technology, Samsung Advanced Institute for Health Sciences & Technology, Samsung Medical Center, Sungkyunkwan University, Seoul, Republic of Korea; ^13^Department of Intelligent Precision Healthcare Convergence, Seoul, Republic of Korea

**Keywords:** Alzheimer’s disease, amyloid beta, positron emission tomography, subcortical vascular cognitive impairment (SVCI), single nucleotide polymorphism (SNP)

## Abstract

**Background:**

The genetic basis of amyloid β (Aβ) deposition in subcortical vascular cognitive impairment (SVCI) is still unknown. Here, we investigated genetic variants involved in Aβ deposition in patients with SVCI.

**Methods:**

We recruited a total of 110 patients with SVCI and 424 patients with Alzheimer’s disease-related cognitive impairment (ADCI), who underwent Aβ positron emission tomography and genetic testing. Using candidate AD-associated single nucleotide polymorphisms (SNPs) that were previously identified, we investigated Aβ-associated SNPs that were shared or distinct between patients with SVCI and those with ADCI. Replication analyses were performed using the Alzheimer’s Disease Neuroimaging Initiative (ADNI) and Religious Orders Study and Rush Memory and Aging Project cohorts (ROS/MAP).

**Results:**

We identified a novel SNP, rs4732728, which showed distinct associations with Aβ positivity in patients with SVCI (*P*_*interaction*_ = 1.49 × 10^–5^); rs4732728 was associated with increased Aβ positivity in SVCI but decreased Aβ positivity in ADCI. This pattern was also observed in ADNI and ROS/MAP cohorts. Prediction performance for Aβ positivity in patients with SVCI increased (area under the receiver operating characteristic curve = 0.780; 95% confidence interval = 0.757–0.803) when rs4732728 was included. Cis-expression quantitative trait loci analysis demonstrated that rs4732728 was associated with *EPHX2* expression in the brain (normalized effect size = −0.182, *P* = 0.005).

**Conclusion:**

The novel genetic variants associated with *EPHX2* showed a distinct effect on Aβ deposition between SVCI and ADCI. This finding may provide a potential pre-screening marker for Aβ positivity and a candidate therapeutic target for SVCI.

## Introduction

Subcortical vascular cognitive impairment (SVCI), the second most prevalent cause of dementia in East Asia, is characterized by extensive cerebral small vessel disease (CSVD) burdens, which include white matter hyperintensities (WMHs) and multiple lacunes (Román et al., 2002). Although amyloid beta (Aβ) deposition is a pathological hallmark of Alzheimer’s disease-related cognitive impairment (ADCI), it frequently co-exists with SVCI, with approximately 30–40% of patients with SVCI having significant brain Aβ depositions, as measured by positron emission tomography (PET) (Lee et al., 2011, 2014; Kang et al., 2021). Previous studies have also demonstrated that Aβ deposition is associated with poor clinical outcomes in patients with SVCI (Kim et al., 2013a; Lee et al., 2014; Ye et al., 2015).

The aberrant deposition of Aβ in ADCI is related to the decreased Aβ clearance; specifically, decreased Aβ clearance can result from impaired microglial function, enzymatic degradation, perivascular Aβ drainage, and the blood–brain barrier (BBB) function (Grimmer et al., 2012; Tarasoff-Conway et al., 2015). We previously revealed that patients with SVCI showed predominant Aβ deposition in the occipital lobe (Jang et al., 2018) and WMHs were associated with Aβ deposition, particularly in posterior brain regions (Noh et al., 2014). Considering that the posterior regions are vulnerable to ischemic injury, the CSVD burden may impaired Aβ clearance by creating a deficiency in perivascular Aβ drainage and in the BBB (Grinberg and Thal, 2010; Zlokovic, 2011). Therefore, the pathobiology of Aβ deposition in patients with SVCI may differ from that of patients with ADCI (Kim et al., 2013b; Lee et al., 2020).

Regarding Aβ deposition in ADCI, genetic factors play an important role; for example, a number of genetic variants, including *APOE* ∈4, have been strongly associated with Aβ deposition in the brain (Morris et al., 2010; Yan et al., 2021). However, to the best of our knowledge, no previous study evaluated the genetic basis of Aβ deposition in SVCI.

In the present study, we aimed to identify genetic variants involved with Aβ deposition using single nucleotide polymorphism (SNP) data from patients with SVCI and ADCI. We hypothesized that there may be SNPs associated with Aβ deposition that are shared and distinct between patients with SVCI and ADCI.

## Materials and methods

### Study participants (discovery data)

We prospectively recruited 110 patients with SVCI and 424 patients with ADCI [284 with amnestic mild cognitive impairment (aMCI) and 140 with AD dementia (ADD)] who underwent Aβ PET at Samsung Medical Center (Seoul, South Korea) between September 2015 and December 2018 and were genotyped using peripheral blood samples in 2019.

Patients with SVCI satisfied the following criteria for SVCI diagnosis: (i) subjective cognitive complaints from either the patient or a caregiver; (ii) objective cognitive impairment below the 16th percentile in any domain, including attention, language, visuospatial, memory, and frontal/executive functions, on the basis of detailed neuropsychological tests (Kang et al., 2003, 2019; Ahn et al., 2010); (iii) significant ischemia on brain magnetic resonance imaging (MRI), defined as periventricular WMH ≥10 mm and deep WMH ≥25 mm, modified from Fazekas’ ischemia criteria, as described in previous studies (Fazekas et al., 1993; Seo et al., 2009), which met the imaging criteria for SVCI proposed by Erkinjuntti et al. (2000); and (iv) focal neurological symptoms or signs.

Patients with aMCI met the following criteria, modified from Peterson’s criteria (Petersen, 2011): (i) normal activities of daily living; (ii) objective memory impairment according to verbal or visual memory tests, which was below the 16th percentile of that in age- and education-matched norms; and (iii) no dementia. Patients with ADD satisfied the core clinical criteria for probable ADD according to the National Institute of Neurological and Communicative Disorders and Stroke and Alzheimer’s Disease and Related Disorders Association (McKhann et al., 2011).

All patients were evaluated through clinical interviews and neurological and neuropsychological examinations. Patients also underwent laboratory tests, including a complete blood count, blood chemistry assessment, vitamin B12, folate evaluation, syphilis serological assessment, and thyroid function test. Brain MRI confirmed the absence of structural lesions, including territorial cerebral infarction, brain tumors, hippocampal sclerosis, and vascular malformations.

All participants provided written informed consent, and the study was approved by the Institutional Review Board of the Samsung Medical Center.

### Genotype data

Peripheral blood samples were genotyped using the Illumina Asian Screening Array BeadChip (Illumina, CA, USA), and SNP markers were analyzed. Quality control (QC) was conducted using PLINK software (version 1.9) (Purcell et al., 2007). Patients were excluded according to the following criteria: (i) call rate <95%, (ii) mismatch between reported and genetically inferred sex, (iii) deviation from each population parameter [5 SD from the sample mean based on the first or second genomic principal components (PCs) of genetic ancestry], and (iv) excess heterozygosity rate (5 SD from the mean). If two patients were related to the second or closer degree, as assessed using KING (Manichaikul et al., 2010), one of the two was excluded. SNPs were excluded based on the following criteria: (i) call rate <98%, (ii) minor allele frequency (MAF) <1%, and (iii) a *P*-value < 10^–6^ in the Hardy-Weinberg equilibrium test. After QC, genome-wide imputation was performed using Minimac4 software and all available reference haplotypes from HRC-r1.1 at the University of Michigan Imputation Server (Howie et al., 2012; Fuchsberger et al., 2015). For post-imputation QC, we excluded SNPs according to the following criteria: (i) poor imputation quality (*r*^2^ ≤ 0.8) and (ii) MAF ≤1%. Among the filtered SNPs, we restricted our analysis to AD-associated SNPs using summary statistics published by the International Genomics of Alzheimer’s Project (IGAP) (Kunkle et al., 2019). IGAP is one of the largest studies (composed of 41,944 AD patients and 21,982 controls), results from which have been validated in a number of subsequent studies. We selected SNPs with genome-wide suggestive associations with AD diagnosis (*P* < 1 × 10^–6^) based on summary statistics from IGAP (Kunkle et al., 2019). Finally, 2,548 SNPs were analyzed in this study.

### Aβ PET acquisition and visual assessment

Amyloid β PET images were obtained using a Discovery STE PET/CT scanner (GE Medical Systems, WI, USA). The PET images were acquired 90 min after an intravenous injection with ^18^F-florbetaben or ^18^F-flutemetamol. The acquisition time was 20 min. Aβ positivity or negativity was determined by well-trained nuclear physicians using visual assessments of florbetaben (Barthel et al., 2011) and flutemetamol (Curtis et al., 2015) PET images. Briefly, positivity for tracer uptake was assessed in four cortical regions (lateral temporal, frontal, parietal, and posterior cingulate cortices) for florbetaben PET and five cortical regions (lateral temporal, frontal, parietal, posterior cingulate cortices, and striatum) for flutemetamol PET. Amyloid PET positivity was defined as having at least one cortical region with evidence of positive uptake.

### Replication data

For the first replication analysis, we used data from individuals enrolled in the Alzheimer’s Disease Neuroimaging Initiative (ADNI)-GO/2 dataset, with available genetic, Aβ PET, and WMH volume data. For the second replication analysis, we used data from Religious Orders Study and Rush Memory and Aging Project (ROS/MAP) cohorts (Bennett et al., 2018). The details of the two cohorts are described in Supplementary File.

### Statistical methods

#### Discovery analysis

We performed two analyses to identify genetic variants associated with Aβ positivity that were shared (the same effect) or distinct (the opposite effect) between patients with SVCI and those with ADCI.

First, to identify shared SNPs, we used a logistic regression model with covariates (including age, sex, education, diagnosis, and the first four PCs of genetic ancestry) expressed as: Aβ positivity = β_0_ + β_1_ age + β_2_ sex + β_3_ education + β_4_ diagnosis (SVCI or ADCI) + β_5_ PC_1_ + β_6_ PC_2_ + β_7_ PC_3_ + β_8_ PC_4_ + β_9_ SNP (additive model, 0, 1, and 2 as the number of minor alleles).

Second, to identify distinct SNPs between SVCI and ADCI, we included the interaction term in the logistic regression model, expressed as: Aβ positivity = β_0_ + β_1_ age + β_2_ sex + β_3_ education + β_4_ diagnosis + β_5_ PC_1_ + β_6_ PC_2_ + β_7_ PC_3_ + β_8_ PC_4_ + β_9_ SNP + β_10_ SNP × diagnosis. The term of interest in this model was the SNP × diagnosis interaction, which identified SNPs with distinct associations with Aβ between SVCI and ADCI. Considering the number of tested SNPs (*n* = 2,548), we defined a *P*-value < 1.96 × 10^–5^ as statistically significant based on the Bonferroni correction (0.05/2,548).

### Replication analysis

Because the ADNI database only recruited patients with ADCI but not with SVCI, we used the WMH volume data, which is a hallmark of SVCI. We used a multivariable logistic regression model, including the WMH volume. To replicate the association of distinct SNPs, we included the interaction term in the logistic regression model, expressed as: Aβ positivity = β_0_ + β_1_ age + β_2_ sex + β_3_ education + β_4_ intracranial volume + β_5_ WMH + β_6_ SNP + β_7_ SNP × WMH. This model evaluates whether the association of SNPs with Aβ positivity differs according to the level of WMH.

Regarding the replication in the ROS/MAP cohorts, we leveraged both amyloid and cerebral vessel pathology data. Aβ positivity was determined using the binarized score of the Consortium to Establish a Registry for Alzheimer’s Disease (negative for none to sparse, positive for moderate to frequent) (Bennett et al., 2006). Cerebral vessel pathology was scored based on the severity of arteriosclerosis, as follows: negative for none to mild and positive for moderate to severe (Nag et al., 2015). To replicate the association of distinct SNPs, we included the interaction term in the logistic regression model, expressed as: Aβ positivity = β_0_ + β_1_ age at death + β_2_ sex + β_3_ education + β_4_ post-mortem interval + β_5_ study (ROS or MAP) + β_6_ cerebral arteriosclerosis + β_7_ SNP + β_8_ SNP × cerebral arteriosclerosis. This model evaluates whether the association of SNPs with Aβ positivity differs according to the presence of cerebral arteriosclerosis. In addition, we evaluated whether SNP interacts with the degree of cerebral amyloid angiopathy on Aβ positivity using the following model: Aβ positivity = β_0_ + β_1_ age at death + β_2_ sex + β_3_ education + β_4_ post-mortem interval + β_5_ study (ROS or MAP) + β_6_ cerebral amyloid angiopathy + β_7_ SNP + β_8_ SNP × cerebral amyloid angiopathy. For the replication analyses, we defined a significance level of *P* < 0.05.

### Functional analysis

We characterized the function of the identified SNPs by leveraging bioinformatics tools. First, we checked whether the MAF of SNPs in our data was similar to that in East Asian populations using the 1000 Genome Project dataset (Sherry et al., 2001). Next, we performed enrichment analysis using HaploReg (version 4.1) and cis-expression quantitative trait loci (cis-eQTL) analysis through the Genotype-Tissue Expression portal (Carithers and Moore, 2015)^1^. Detailed description of the functional analysis is provided in Supplementary File.

### Prediction of Aβ positivity using the newly identified SNPs

To test the clinical utility of the newly identified SNPs, we developed multivariable logistic models to predict Aβ positivity in each individual. We performed receiver operating characteristic curve analysis and measured the area under the receiver operating characteristic curve (AUC). As an internal validation, we conducted a 10-fold cross-validation with 100 repeats. Data are reported as the mean AUC and 95% confidence interval (CI).

## Results

### Clinical characteristics of the study participants

Table 1 shows the baseline demographics of the discovery and replication datasets. In the discovery dataset, 67.0% of the patients with ADCI and 35.5% of those with SVCI showed positive results for Aβ deposition in the brain.

**TABLE 1:** Demographics of study participants.

	Discovery data			Replication data	
Demographics	Total (*n* = 534)	SVCI (*n* = 110)	ADCI (*n* = 424)	ADNI (*n* = 680)	ROS/MAP (*n* = 1,019)
Age, years, mean (SD)	74.8 (7.8)	77.8 (7.2)	74.1 (7.7)	74.0 (7.2)	89.0 (6.5)
Female, *n* (%)	309 (57.9)	78 (70.9)	231 (54.5)	307 (45.1)	668 (65.5)
Education, year, mean (SD)	10.4 (5.2)	8.1 (5.6)	11.0 (5.0)	16.25 (2.6)	16.3 (3.6)
*APOE* ∈4 (0/1/2), *n*	304/182/48	81/27/2	223/155/46	369/248/63	771/233/15
Aβ positivity, *n* (%)	323 (60.5)^a^	39 (35.5)^a^	284 (67.0)^a^	381 (56.0)^b^	686 (67.3)^c^
WMH, mL, mean (SD)^d^	–	–	–	7.64 (10.43)	–
Presence of cerebral arteriolosclerosis, *n* (%)^e^	–	–	–	–	328 (32.1)

Aβ positivity was determined using either.

^a^Visual assessment for each PET tracers or ^b^a cut-off of 1.11 or ^c^CERAD score (positive, if moderate to frequent neuritic plaques were found in one or more neocortices).

^d^WMH volume was estimated using an automated imaging procedure, as described in the ADNI website (https://adni.loni.usc.edu/data-samples/data-types/mri/).

^e^Presence of cerebral arteriosclerosis was determined if moderate to severe arteriosclerosis were found.

Aβ, amyloid beta; ADCI, Alzheimer’s disease-related cognitive impairment; SD, standard deviation; SVCI, subcortical vascular cognitive impairment; WMH, white matter hyperintensity.

### Discovery analysis

Analysis of Aβ-associated SNPs that were shared between patients with SVCI and those with ADCI revealed 23 SNPs on chromosome 19 (*P* < 1.961 × 10^–5^) (Table 2). These significant SNPs were located within a 500-kb region surrounding the *APOE* gene and they lost genome-wide significance when adjusted for the *APOE* ∈4 allele.

**TABLE 2:** Aβ-associated SNPs that are shared between SVCI and ADCI.

SNP	CHR:BP	OR	Beta	*P*-value	*P*-value^a^
*APOE* ∈4	19	6.55	1.879	7.45 × 10^–19^	
rs73050216	19: 45,367,502	0.44	-0.820	2.04 × 10^–17^	0.007
rs12610605	19: 45,370,838	0.41	-0.891	3.50 × 10^–16^	0.028
rs34278513	19: 45,378,144	3.22	1.169	1.96 × 10^–12^	0.256
rs412776	19: 45,379,516	4.05	1.398	4.78 × 10^–13^	0.220
rs3865427	19: 45,380,961	3.59	1.278	1.34 × 10^–13^	0.200
rs6859	19: 45,382,034	2.14	0.760	3.31 × 10^–96^	0.874
rs3852860	19: 45,382,966	3.89	1.358	3.94 × 10^–52^	0.018
rs3852861	19: 45,383,061	3.89	1.358	8.34 × 10^–47^	0.018
rs71352237	19: 45,383,079	4.09	1.408	5.96 × 10^–15^	0.124
rs34224078	19: 45,383,115	4.09	1.408	5.35 × 10^–15^	0.124
rs35879138	19: 45,383,139	4.09	1.408	5.17 × 10^–15^	0.124
rs157580	19: 45,395,266	0.42	-0.867	1.21 × 10^–101^	0.334
rs59007384	19: 45,396,665	3.28	1.187	1.97 × 10^–486^	0.524
rs405697	19: 45,404,691	0.39	-0.941	2.26 × 10^–50^	0.335
rs10119	19: 45,406,673	6.88	1.928	1.21 × 10^–342^	0.084
rs440446	19: 45,409,167	0.39	-0.941	2.30 × 10^–67^	0.594
rs439401	19: 45,414,451	0.38	-0.967	3.55 × 10^–79^	0.328
rs10414043	19: 45,415,713	6.35	1.848	1.15 × 10^–522^	0.153
rs7256200	19: 45,415,935	6.35	1.848	1.80 × 10^–520^	0.153
rs584007	19: 45,416,478	0.38	-0.967	1.06 × 10^–82^	0.356
rs12721046	19: 45,421,254	6.48	1.868	1.05 × 10^–421^	0.150
rs56131196	19: 45,422,846	6.55	1.879	1.96 × 10^–454^	0.125
rs157595	19: 45,425,460	2.41	0.879	3.76 × 10^–101^	0.374

*P*-value was calculated using the logistic regression analysis.

^a^*P*-value was calculated using logistic regression analysis with adjustment for the APOE ∈4 allele.

CHR, chromosome; BP, base pair; OR, odds ratio; SNP, single-nucleotide polymorphism.

**FIGURE 1:**
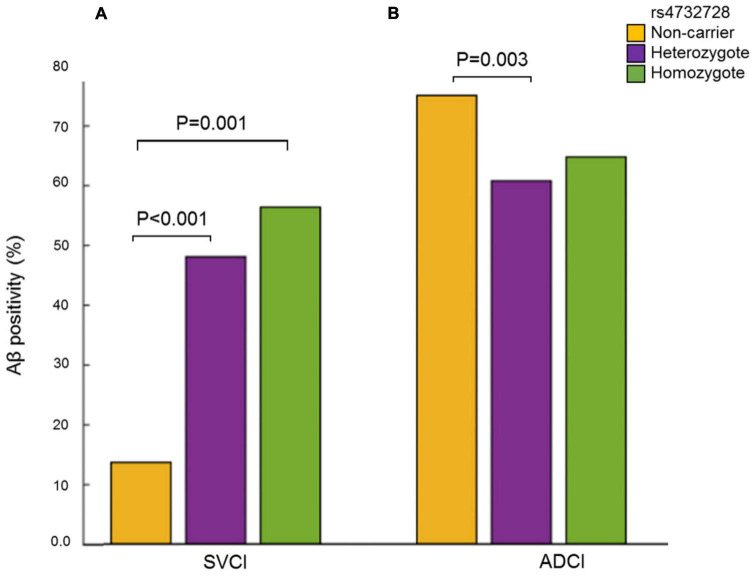
Frequency of Aβ positivity according to carrier status of the rs4732728. **(A)** SVCI. **(B)** ADCI. *P*-values were calculated using the Chi-square test. Aβ, amyloid beta; ADCI, Alzheimer’s disease-related cognitive impairment; SVCI, subcortical vascular cognitive impairment.

**FIGURE 2:**
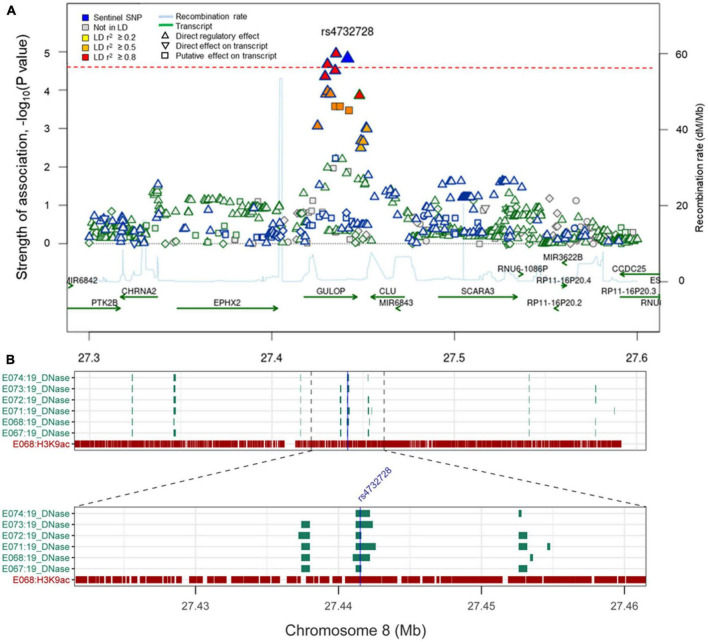
**(A)** Regional association plot of rs4732728. The red dotted line indicates *P*-value threshold (1.96 × 10^– 5^). *P*-values were calculated using the logistic regression with the interaction term (SNP × diagnosis). The figure was modified from the SNiPA (single-nucleotide polymorphism annotator) (https://snipa.helmholtz-muenchen.de/snipa3). **(B)** Chromatin state of rs4732728 in brain tissues. Brain angular gyrus (E067), brain anterior caudate (E068), brain hippocampus middle (E071), brain inferior temporal lobe (E072), brain dorsolateral prefrontal cortex (E073), and brain substantia nigra (E074). The figure was based on the Roadmap Epigenomics (https://egg2.wustl.edu/roadmap/web_portal).

The analysis of Aβ-associated SNPs that were distinct between patients with SVCI and those with ADCI revealed one significant SNP on chromosome 8, rs4732728 (β = 1.58, *P* = 1.49 × 10^–5^; Table 3). A similar result was observed after adjusting for the *APOE* ∈4 allele (β = 1.60, *P* = 7.19 × 10^–5^). Subgroup analyses based on the diagnosis (SVCI or ADCI) showed that rs4732728 was associated with a 4.58-fold higher risk of Aβ deposition in SVCI [odds ratio (OR) = 4.58, *P* = 8.04 × 10^–5^] and a 1.32-fold lower risk of Aβ deposition in ADCI (OR = 0.76, *P* = 0.01) (Figure 1). In the regional association plot of rs4732728 (Figure 2), SNPs in high linkage disequilibrium (LD; *r*^2^ > 0.8) also had a significant interaction with SVCI on Aβ deposition (Table 3).

**TABLE 3:** Aβ-associated SNPs that are distinct between SVCI and ADCI.

SNP	CHR:BP	EA	Beta (*P*-value)	
			SNP	SNP × SVCI
rs4732728	8:27,441,521	C	−0.39 (0.012)	1.58 (1.49 × 10^–5^)
rs1316801	8:27,429,228	C	−0.41 (0.008)	1.43 (4.40 × 10^–5^)
rs7831810	8:27,430,506	A	−0.406 (0.010)	1.56 (2.12 × 10^–5^)
rs10780145	8:27,434,722	C	−0.426 (0.007)	1.54 (3.07 × 10^–5^)
rs4352801	8:27,435,201	T	−0.477 (0.002)	1.62 (1.12 × 10^–5^)
rs6983452	8:27,448,028	C	−0.346 (0.025)	1.36 (1.37 × 10^–4^)

Beta coefficient and P-value were calculated using the logistic regression analysis. A, adenine; BP, base pair; C, cytosine; CHR, chromosome; EA, effective allele; T, thymine; SNP, single-nucleotide polymorphism.

### Replication analyses

In the ADNI cohort, there was a significant interaction between rs4732728 and the level of WMH on Aβ positivity (β = 0.531, *P* = 0.02), with the effect being in the same direction as that in the discovery analysis. The positive association between rs4732728 and Aβ positivity increased as the WMH volume increased.

In the ROS/MAP cohorts, there was a significant interaction between rs4732728 and the presence of cerebral arteriosclerosis on Aβ positivity (β = 0.44, *P* = 0.03), with the effect being in the same direction as that in the discovery analysis. The positive association between rs4732728 and Aβ positivity increased in the presence of cerebral arteriosclerosis. In addition, there was a significant interaction between rs4732728 and the degree of cerebral amyloid angiopathy on Aβ positivity (β = 0.28, *P* = 0.02).

### Functional characterization of rs4732728

The frequency of the effective allele (cytosine) of rs4732728 in the discovery dataset was 0.333, and that of the two replication datasets was 0.593 [cognitively unimpaired (CU) subjects of ADNI (*n* = 203)] and 0.577 [CU subjects of ROS/MAP (*n* = 359)], respectively. This was in accordance with the previously reported frequencies of 0.382 and 0.580 for East Asian and European populations (The 1000 Genomes Project Consortium et al., 2015), indicating that the samples used in this study represent each ancestry populations.

We characterized the function of the novel SNP rs4732728 using bioinformatics tools. rs4732728 is located in the intron of gulonolactone (L-) oxidase (*GULOP*). HaploReg based on ChromHMM annotated rs4732728 as a DNase I hypersensitive site in brain tissues (hippocampus middle, substantia nigra, anterior caudate, inferior temporal lobe, angular gyrus, and dorsolateral prefrontal cortex), indicating that this SNP is in an accessible chromatin region. We also found positive results for the presence of the histone modification mark H3K9ac (active promoter state) of rs6983452 (SNP of high LD with rs4732728), indicating acetylation of the 9th lysine residue of the histone H3 protein, in the anterior caudate and angular gyrus (Figure 2).

**FIGURE 3:**
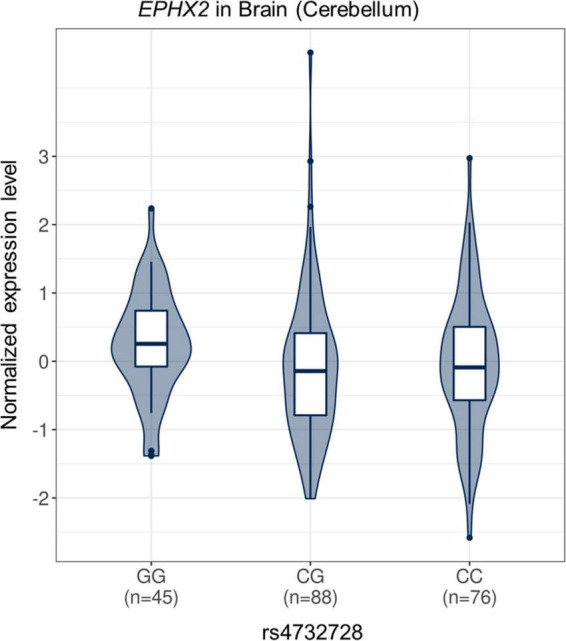
Violin plot of *EPHX2* expression according to rs4732728 genotype. The allelic effect of rs4732728 on normalized *EPHX2* gene expression levels are shown. The figure was based on the Genotype-Tissue Expression database (http://gtexportal.org). C, cytosine; G, guanine.

In the cis-eQTL analysis using the GTEx database, the rs4732728 and additional five high LD SNPs (rs1316802, rs7831810, rs10780145, rs4352801, and rs6983452) showed significant cis-eQTL effects on epoxide hydrolase 2 (*EPHX2*) in the brain, and a greater dosage of SNPs decreased the expression of *EPHX2* [rs4732728: normalized effect size (NES) = −0.182, *P* = 0.005; rs1316801: NES = −0.181, *P* = 0.006; rs7831810: NES = −0.191, *P* = 3.8 × 10^–3^; rs10780145: NES = −0.192, *P* = 3.5 × 10^–3^; rs4352801: NES = −0.181, *P* = 0.008; rs6983452: NES = −0.182, *P* = 4.9 × 10^–3^; Figure 3). No SNP showed significant cis-eQTL effects on *GULOP* in the brain.

### Prediction of Aβ positivity in SVCI and ADCI

**FIGURE 4:**
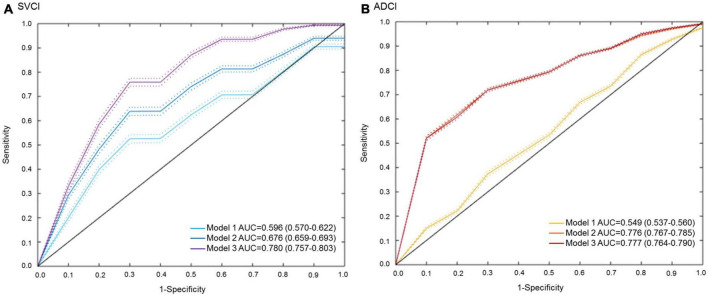
ROC curves for the prediction of Aβ positivity. **(A)** SVCI. **(B)** ADCI. Solid lines indicate the mean AUC and dotted lines indicate the 95% CIs of the AUC. Each model was developed by multivariable logistic regression. Model 1: Aβ positivity ∼ clinical factors (sex, age, and education). Model 2: Aβ positivity ∼ clinical factor + *APOE* ∈4. Model 3: Aβ positivity ∼ clinical factor + *APOE* ∈4 + rs4732728. Aβ, amyloid beta; ADCI, Alzheimer’s disease-related cognitive impairment; AUC, area under the receiver operating characteristic curve; CI, confidence interval; ROC, receiver operating characteristic; SVCI, subcortical vascular cognitive impairment.

To test the clinical utility of rs4732728, we developed logistic models to predict Aβ positivity in SVCI and ADCI. In the cross validation, the model including clinical factors (age, sex, and education) and the *APOE* ∈4 allele showed an AUC of 0.676 (95% CI = 0.659–0.693) and 0.776 (95% CI = 0.767–0.785) in SVCI and ADCI, respectively, (Model 2 of Figure 4). When the model included rs4732728 (Model 3 of Figure 4), a significant increase in the prediction performance was observed in SVCI (AUC = 0.780, 95% CI = 0.757–0.803) but not in ADCI (AUC = 0.777, 95% CI = 0.764–0.790; Figure 4).

## Discussion

In the present study, we identified a novel SNP showing a distinct effect on Aβ deposition between SVCI and ADCI. Our major findings are as follows: First, rs4732728 was associated with increased Aβ positivity in SVCI but decreased Aβ positivity in ADCI. The interaction between rs4732728 and CSVD markers on Aβ deposition was replicated in independent ADNI and ROS/MAP cohorts. Second, the functional analysis revealed that rs4732728 was associated with decreased expression levels of *EPHX2* in the brain. Finally, rs4732728 contributed to increased accuracy in the prediction of Aβ positivity in patients with SVCI.

We observed that variants in the *APOE* locus were associated with increased Aβ positivity not only in patients with ADCI but also in those with SVCI. This is accordance with previous study where *APOE* ∈4 allele increases the risk of Aβ deposition in patients with SVCI (Kim et al., 2013b). Notably, we identified a novel locus showing distinct associations with Aβ positivity in the patient groups. Specifically, patients with SVCI who carried a minor allele (cytosine) of rs4732728 showed an increased risk of Aβ positivity, whereas those with ADCI showed a decreased risk of Aβ positivity. Similar findings were observed in two other independent cohorts comprising participants of European ancestry. This indicates that the identified SNPs may be functional in populations of various ancestries. In addition, different CSVD markers were used among the three datasets; we used WMH volumes in the ADNI dataset, arteriosclerosis severity in the ROSMAP dataset, and the diagnosis of SVCI in the discovery dataset. Nonetheless, the findings were consistent in various measures of vascular pathologies.

The eQTL analysis revealed that the minor allele (cytosine) of rs4732728 was associated with decreased expression levels of *EPHX2* in the brain, suggesting that this gene may be a link between rs4732728 and Aβ deposition. *EPHX2* encodes an enzyme, epoxide hydrolase, which binds to specific epoxides and converts them to the corresponding diols (Morisseau and Hammock, 2013). In a previous study of AD, the expression of microsomal epoxide hydrolase was increased in the hippocampal tissues of patients with AD (Liu et al., 2006). Furthermore, genetic deletion of soluble epoxide hydrolase was found to reduce Aβ deposition and delay progression of AD in transgenic mice (Lee et al., 2019; Chen et al., 2020; Ghosh et al., 2020). In a previous study of cerebrovascular disease, decreased levels of epoxide hydrolase were associated with increased neuronal survival after ischemic injury via changes in the levels of epoxyeicosatrienoic acids (Koerner et al., 2007). A recent study also demonstrated significant association of *EPHX2* genetic variation with cerebrovascular disease (Zhu et al., 2022). The findings of these previous studies suggest that patients with the minor allele (cytosine) of rs4732728 and low levels of *EPHX2* would be more resistant to Aβ deposition and ischemic injury than those with the major allele (guanine). These results may explain the distinct associations of rs4732728 with Aβ positivity in patients with SVCI. In SVCI patient with the minor allele (cytosine) of rs473278, Aβ deposition may contribute to cognitive impairment because white matter changes may be less pathogenic to these patients. In contrast, in SVCI patients with the major allele (guanine) of rs4732728, white matter changes are sufficient to cause cognitive impairment since these patients were more susceptible to ischemic injury. Further genomic studies are necessary to elucidate the biological mechanism underlying the distinct actions of rs4732728 on Aβ deposition in patients with SVCI and ADCI.

Identifying patients with SVCI with brain Aβ deposition is important in predicting the prognosis and successful intervention, with the expectation that future treatments may target Aβ. However, currently available diagnostic tools for measuring Aβ are either invasive (cerebrospinal fluid examination) or expensive (PET) (Fargo et al., 2016). In the present study, we demonstrated that genetic data (*APOE* ∈4 and rs4732728) from blood with clinical information could predict Aβ positivity in patients with SVCI. Considering that the rate of Aβ positivity in our SVCI cohort was 35.5%, 275 patients would be required to perform Aβ PET in order to obtain 100 patients with Aβ deposition. In contrast, when we applied the prediction model including rs4732728, the number of individuals that would need to undergo Aβ PET was reduced by 58%. This result suggests that rs4732728 may play a role as a potential pre-screening marker for Aβ positivity in patients with SVCI. However, this result needs to be validated using independent datasets.

In addition to rs473728, as a pre-screening marker for Aβ positivity in SVCI, our results support the possible therapeutic target of *EPHX2* for cerebrovascular disease (Zuloaga et al., 2015). Because drugs that control epoxide hydrolase level are available, the clinical trial can be conducted for SVCI in the future.

The strength of our study is that we performed a genetic study in thoroughly phenotyped patients with ADCI and SVCI using Aβ PET and structural MRI. However, this study has several limitations. First, the sample size was relatively small compared to that of recent genome-wide association studies. Second, the statistical significance level in the replication dataset was small compared to that in the discovery dataset. The difference might result from the heterogeneities between the dataset in terms of pathology measures (Aβ and CSVD), clinical demographics, and genetic backgrounds. Nevertheless, the similar observations in the two independent datasets and the biological relevance of the identified SNPs both strengthen the validity of our findings. Third, we used candidate SNPs that have previously been identified in genome-wide association studies for AD diagnosis. Future whole-genome analyses using larger datasets may identify additional genetic variants that were not tested in this study. Fourth, we could not investigate the biological mechanism underlying the distinct effects of the identified SNPs on Aβ between patients with SVCI and ADCI. Future functional studies using gene editing are necessary to elucidate the underlying mechanisms. Fifth, Aβ PET could not discriminate between different Aβ isoforms. As Aβ shows parenchymal or vascular deposition depending on dominance of Aβ42 or Aβ40 (Yamada, 2012), measuring different Aβ isoforms might be helpful in this study. Finally, as alternative pathomechanisms such as tau, neuroinflammation, and oxidative stress also contribute to both ADCI and SVCI (Román et al., 2002; Gong et al., 2018), mechanisms other than Aβ should be evaluated in the future.

## Conclusion

In summary, we identified novel SNPs that showed a distinct effect on Aβ deposition between SVCI and ADCI. The identified SNP showed an additive predictive value for Aβ positivity in patients with SVCI and showed an association with expression of the *EPHX2* gene. This finding may provide a potential pre-screening marker for Aβ positivity and a candidate therapeutic target for SVCI.

## Data availability statement

The original contributions presented in this study are included in the article/Supplementary material, further inquiries can be directed to the corresponding authors.

## Ethics statement

This studies was involving human participants were reviewed and approved by the Samsung Medical Center. The patients/participants provided their written informed consent to participate in this study.

## Author contributions

H-RK, H-HW, and SS contributed to the study conception and design. H-RK, JK, HJ, DN, HK, H-HW, and SS performed the material preparation and data collection. H-RK, S-HJ, BK, JPK, SK, KN, H-HW, and SS performed the data analysis. H-RK wrote the first draft of the manuscript. All authors contributed to manuscript revision, read, and approved the final manuscript.

## Alzheimer’s Disease Neuroimaging Initiative

Data used in preparation of this article were obtained from the Alzheimer’s Disease Neuroimaging Initiative (ADNI) database (adni.loni.usc.edu). As such, the investigators within the ADNI contributed to the design and implementation of ADNI and/or provided data but did not participate in the analysis or writing of this manuscript. A complete listing of ADNI investigators can be found at http://adni.loni.usc.edu/wp-content/uploads/how_to_apply/ADNI_Acknowledgement_List.pdf.

## Footnotes

1
http://gtexportal.org
