# Supplementary material for: Identifying genetic variants for amyloid β in subcortical vascular cognitive impairment
Source: Front Aging Neurosci. 2023 Apr 18;15:1160536. doi: 10.3389/fnagi.2023.1160536 (PMC10151714; doi:10.3389/fnagi.2023.1160536)
Supplement: Supplementary file 1 [file Data_Sheet_1.docx]

**Identifying genetic variants for amyloid β in subcortical vascular cognitive impairment**

**Supplementary materials**

**Replication data**

**ADNI cohorts**

For the first replication analysis, we used data from individuals enrolled in the Alzheimer’s Disease Neuroimaging Initiative (ADNI)-GO/2 dataset, with available genetic, Aβ PET, and WMH volume data. The ADNI was launched in 2003 as a public-private partnership led by Principal Investigator Michael W. Weiner, MD. Its primary goal has been to test whether serial MRI, PET, other biological markers, and clinical and neuropsychological assessments can be combined to measure the progression of MCI and early AD. Data of 680 patients of European ancestry (203 cognitively unimpaired [CU], 288 with MCI, and 189 with ADD) were used in the replication analysis. Detailed diagnostic criteria are described in the ADNI website (http://www.adni-info.org). The study protocol was approved by the Institutional Review Board of each participating ADNI site (http://adni.loni.usc.edu/wp-content/uploads/how_to_apply/ADNI_Acknowledgement_List.pdf), and participants provided written informed consent at the time of enrollment.

Participants’ samples were genotyped using the Illumina HumanOmni BeadChip (Illumina, CA, USA). We performed quality control and imputation for the genetic data as described in our previous study (Kim et al., 2020). For assessing consistency between the Korean and ADNI European cohorts, we used a minor allele in the Korean cohort as the effective allele in subsequent analysis.

For measuring Aβ levels, we used summarized data of ^18^F-florbetapir PET, generated from the University of California, Berkeley (version 2017.11.14). We used the averaged value of the Aβ standardized uptake ratio of the four brain regions (frontal, cingulate, lateral parietal, and lateral temporal cortex) for calculating the global Aβ deposition value. Aβ positivity was determined using a cutoff of 1.11 based on previous studies (Clark et al., 2011;Joshi et al., 2012).

We used the summarized data of WMH volume (mL) generated from the University of California, Davis (version 2019.4.30). The mean time interval between WMH and PET data was 96 days (standard deviation, 215). The WMH volume was estimated using an automated imaging procedure. A detailed description of the procedure is provided on the ADNI website (https://adni.loni.usc.edu/data-samples/data-types/mri/). We log-transformed the WMH volume for the data normality.

**ROS/MAP cohorts**

For the second replication analysis, we used data from Religious Orders Study and Rush Memory and Aging Project (ROS/MAP) cohorts (Bennett et al., 2018).

ROS is comprised of older Catholic priests, nuns, and monks throughout the USA. MAP recruited older lay persons from the greater Chicago area. Both studies involved detailed annual cognitive and clinical evaluations and brain autopsy. Participants provided informed consent, signed an Anatomic Gift Act, and a repository consent to allow their data and biospecimens to be repurposed. The studies were approved by an Institutional Review Board of Rush University Medical Center. Data of 1019 subjects, whose final clinical diagnosis were either CU (n=359), MCI (n=270), or AD (n=390), were used. Clinical diagnosis was determined at the time of death by a neurologist with expertise in dementia using all available clinical data but blinded to postmortem data.

The presence of Aβ was determined using Consortium to Establish a Registry for Alzheimer’s Disease (CERAD) score, a semiquantitative measure of neuritic plaques consisting of the following score: no neuritic plaque (C0), CERAD score sparse (C1), CERAD score moderate (C3), CERAD score frequent (C4). A CERAD neuropathologic diagnosis of AD (Aβ positivity) required moderate (C3) or frequent neuritic plaques (C4) in one or more neocortical regions (Bennett et al., 2006).

Assessment for cerebral vessel pathology was also systemically conducted. For the small vessels, arteriolosclerosis was assessed based on concentric hyalinized thickening of the walls of small arterioles in anterior basal ganglia. Cerebral amyloid angiopathy was assessed based on evaluation of paraffin-embedded sections of four neocortical regions (midfrontal, middle temporal, inferior parietal, and occipital) immunostained for beta-amyloid (antibody, Covance Labs, Dako, or Elan Pharmaceuticals). Severity was graded on a semiquantitative scale consisting of the following categories: none (0), mild (1), moderate (2), and severe (3), as previously described (Nag et al., 2015).

**Functional analysis**

We characterised the function of the identified SNPs by leveraging bioinformatics tools. First, we checked whether the MAF of SNPs in our data was similar to that in East Asian populations using the 1000 Genome Project dataset (Sherry et al., 2001). Next, we performed enrichment analysis using HaploReg (version 4.1) to infer the chromatin state of the identified SNPs (Ward and Kellis, 2016). Twelve histone modification marks (H3K4me1, H3K4me2, H3K4me3, H3K9ac, H3K27ac, H4K20me1, H3K79me2, H3K36me3, H3K9me3, H3K27me3, H2A.Z, and DNase) were used to characterize chromatin states by ChromHMM for seven brain tissues (hippocampus middle, substantia nigra, anterior caudate, cingulate gyrus, inferior temporal lobe, angular gyrus, and dorsolateral prefrontal cortex). ChromHMM is based on a multivariate hidden Markov model that integrates multiple chromatin datasets, such as ChIP-seq data of various histone modification markers to characterise chromatin states (Ernst and Kellis, 2017). Finally, to evaluate the genotype-specific expression of the identified SNPs in human brain tissues, we performed cis-expression quantitative trait loci (cis-eQTL) analysis through the Genotype-Tissue Expression portal (http://gtexportal.org) (Carithers and Moore, 2015). We report genes with significant changes in expression in brain tissues by the genotype of the SNP (*P* < 0.05).

**References**

Bennett D, Schneider J, Arvanitakis Z et al (2006) Neuropathology of older persons without cognitive impairment from two community-based studies. *Neurology*. 66,1837-1844.

Bennett DA, Buchman AS, Boyle PA, Barnes LL, Wilson RS, Schneider JA (2018). Religious orders study and rush memory and aging project. *J Alzheimers Dis.* 64,S161-S189.

Carithers LJ, Moore HM (2015) The genotype-tissue expression (GTEx) project. *Biopreserv Biobank.* 13,307-308.

Clark CM, Schneider JA, Bedell BJ et al (2011) Use of florbetapir-PET for imaging β-amyloid pathology. *JAMA.* 305,275-283.

Ernst J, Kellis M (2017) Chromatin-state discovery and genome annotation with ChromHMM. *Nat Protoc.* 12, 2478-2492.

Joshi AD, Pontecorvo MJ, Clark CM et al (2012) Performance characteristics of amyloid PET with florbetapir F 18 in patients with Alzheimer's disease and cognitively normal subjects. *J Nucl Med*. 53,378-84.

Kim H-R, Lee T, Choi JK, Jeong Y (2020) Genetic variants beyond amyloid and tau associated with cognitive decline: A cohort study. *Neurology.* 95,e2366-2377.

Nag S, Yu L, Capuano AW et al (2015) Hippocampal sclerosis and TDP‐43 pathology in aging and Alzheimer disease. *Ann Neurol.* 77,942-952.

Sherry ST, Ward M-H, Kholodov M et al (2001) dbSNP: the NCBI database of genetic variation. *Nucleic Acids Res.* 29,308-311.

Ward LD, Kellis M (2016) HaploReg v4: systematic mining of putative causal variants, cell types, regulators and target genes for human complex traits and disease. Nucleic Acids Res. 44, D877-881.
